# Dyslipidemia in Renal Transplant Recipients Treated With Cyclosporine A

**DOI:** 10.5812/numonthly.14167

**Published:** 2013-11-13

**Authors:** Mariusz Niemczyk

**Affiliations:** 1 Department of Immunology, Transplant Medicine and Internal Diseases, Medical University of Warsaw, Warsaw, Poland

**Keywords:** Dyslipidemia, Cyclosporine A, Kidney Transplantation

***Dear Editor,***

Death with a functioning graft is the most common reason for renal transplant loss, and cardiovascular disease is the leading cause of mortality. Dyslipidemia is a risk factor for cardiovascular disease in the general population, and is common after renal transplantation. Despite the fact that the relationship between dyslipidemia and cardiovascular mortality has not been proven in transplant recipients, dyslipidemia is considered as a condition that should be aggressively treated in this population (1, 2). Hosseini et al. (3), searching for the correlation between dyslipidemia and both trough level (C0) and two hour post dose level (C2) of Cyclosporine A (CsA), retrospectively analysed a relatively large group of kidney transplant recipients. The problem is that the results of Hosseini et al. are not fully comparable to other reports, as different definitions of dyslipidemia were used in particular papers, e.g. Hosseini et al. considered low-density lipoprotein (LDL) level as high at 130 mg/dL, while others reported LDL level exceeding 100 mg/dL as too high (1, 2). Additionally, many key factors were omitted in the analysis of Hosseini et al., e.g. data on lipid-lowering medications. Finally, in practice, it is rather of low importance whether dyslipidemia correlates with CsA levels. What is important is the awareness of physicians that therapy with CsA leads to increased serum lipid concentrations (4, 5), as well as the universal screening for dyslipidemia carried out in each renal transplant recipient, and effective treatment of this condition.

## References

[1] Riella LV, Gabardi S, Chandraker A (2012). Dyslipidemia and its therapeutic challenges in renal transplantation.. Am J Transplant..

[2] Kumar R, Brar J, Yacoub R, Khan T, Zachariah M, Venuto R (2012). Assessment of cardiovascular risk factors after renal transplantation: a step towards reducing graft failure.. Transplant Proc..

[3] Hosseini MS, Rostami Z, Einollahi B (2013). Dyslipidemia after kidney transplantation and correlation with cyclosporine level.. Nephro Urol Mon..

[4] Deleuze S, Garrigue V, Delmas S, Chong G, Swarcz I, Cristol JP (2006). New onset dyslipidemia after renal transplantation: is there a difference between tacrolimus and cyclosporine?. Transplant Proc..

[5] Jiang Y, Xie XB, Peng LK, Peng FH, Lan GB, Wang Y (2011). Dyslipidemia in human kidney transplant recipients receiving cyclosporine and tacrolimus is associated with different expression of CD36 on peripheral blood monocytes.. Transplant Proc..

